# Evolution in Australasian Mangrove Forests: Multilocus Phylogenetic Analysis of the *Gerygone* Warblers (Aves: Acanthizidae)

**DOI:** 10.1371/journal.pone.0031840

**Published:** 2012-02-14

**Authors:** Árpád S. Nyári, Leo Joseph

**Affiliations:** 1 Department of Ecology and Evolutionary Biology, Biodiversity Institute, The University of Kansas, Lawrence, Kansas, United States of America; 2 Australian National Wildlife Collection, Commonwealth Scientific and Industrial Research Organisation - Ecosystem Sciences, Canberra, Australian Capital Territory, Australia; Smithsonian Institution National Zoological Park, United States of America

## Abstract

The mangrove forests of Australasia have many endemic bird species but their evolution and radiation in those habitats has been little studied. One genus with several mangrove specialist species is *Gerygone* (Passeriformes: Acanthizidae). The phylogeny of the Acanthizidae is reasonably well understood but limited taxon sampling for *Gerygone* has constrained understanding of its evolution and historical biogeography in mangroves. Here we report on a phylogenetic analysis of *Gerygone* based on comprehensive taxon sampling and a multilocus dataset of thirteen loci spread across the avian genome (eleven nuclear and two mitochondrial loci). Since *Gerygone* includes three species restricted to Australia's coastal mangrove forests, we particularly sought to understand the biogeography of their evolution in that ecosystem. Analyses of individual loci, as well as of a concatenated dataset drawn from previous molecular studies indicates that the genus as currently defined is not monophyletic, and that the Grey Gerygone (*G. cinerea*) from New Guinea should be transferred to the genus *Acanthiza*. The multilocus approach has permitted the nuanced view of the group's evolution into mangrove ecosystems having occurred on multiple occasions, in three non-overlapping time frames, most likely first by the *G. magnirostris* lineage, and subsequently followed by those of *G. tenebrosa* and *G. levigaster*.

## Introduction

Among the members of the primarily Australo-Papuan passerine family Acanthizidae, the genus *Gerygone* Gould, 1841 is the most geographically widespread. Its 19 currently recognized member species occur in Australia, New Guinea, New Zealand, Pacific Islands, Indonesia and south-east Asia as well as on many offshore islands. One species, *G. sulphurea*, is found north of Wallace's Line from Thailand to the Philippines, and *G. insularis* of Lord Howe Island became extinct following predation by introduced rats in the early 19^th^ century [1]. All species of *Gerygone* are small, relatively drab, and forage arboreally. Habitats range from closed canopy moist forests to open arid zone woodlands, and at least three species (*G. magnirostris*, *G. tenebrosa*, *G. levigaster*) occur predominantly in coastal mangrove forests, and another, *G. chloronota*, enters them in Australia as well [1]–[3]. Given their diverse biogeographic and ecological patterns, gerygones are among the groups best-suited for elucidating the origin of Australia's rich mangrove avifauna [2]–[5]. To date, the inclusion of Australasian mangrove specialist bird species in molecular phylogenetic studies has been incidental rather in relevant work [6]–[7]. *Gerygone* provides an ideal group with which to redress this. They are an ideal group with which to apply molecular phylogenetics to the testing of hypotheses that have been advanced for evolution of mangrove specialist birds in the region [2]–[5].

Despite Ford's (1986) pioneering attempt to analyze *Gerygone* phylogenetically, the conservative morphology of the group has inhibited development of a comprehensive phylogenetic framework. This in turn has complicated interpretations of biogeographic patterns. A recent phylogenetic study of the largest radiation of Australasian songbirds, the Meliphagoidea [8], was the first molecular analysis of acanthizids that included *Gerygone*. The eight species of *Gerygone* analysed there comprised a monophyletic group, which, together with the monotypic Fernwren *Oreoscopus gutturalis*, was basal to all other acanthizids. Support for the monophyly of the eight species was high but relationships within the genus were not well resolved.

Several molecular phylogenetic studies have now documented the importance of island radiations in diversification of continental avifaunas [9]–[11]. They have led to the conclusion that islands are not necessarily evolutionary dead ends, but rather that they can be sources of biological diversity for mainland groups through back-colonization events. By analogy, the role of mangrove forests as ecological islands for closed-canopy-dwelling birds, especially during Australia's long history of aridification [12], might also be tested.

Here we explore the evolution of mangrove-inhabiting species of *Gerygone*. As well as using established mitochondrial DNA-based methodologies, we also explore the question of whether additional resolving power might be brought to the question by way of a multilocus dataset. This approach reflects two now well-established observations: that individual gene trees can differ from the true species tree, and that these datasets offer richer windows into the evolutionary history of lineages than studies based on mitochondrial DNA (mtDNA) [11]–[20]. Gene tree – species tree discordances can result from stochastic sorting of ancestral polymorphisms, or varying degrees of gene flow following lineage-splitting events at different depths within the phylogenetic history of a group of organisms [21]–[23]. Reliable detection and discrimination of all of these confounding processes calls for increased complexity and thoroughness of model-based phylogenetic estimations from multilocus datasets. These range from individual gene tree analysis, concatenation and partitioning of an entire multilocus dataset, to Bayesian Estimation of Species Tree methods. The latter estimates the joint posterior distribution of gene trees for each locus and uses that to approximate the Bayesian posterior distribution of the species tree based on coalescent theory [23], [24]. The implications of these methodological advances are far reaching. Anomalous gene trees [23] are known to be quite common, particularly in groups that have seen rapid bursts of speciation [10].

Accordingly, we here use comprehensive taxon sampling and an analysis of sequence data derived from 13 loci spread across the avian nuclear and mitochondrial genomes to test monophyly of the acanthizid genus *Gerygone* as well as the relationships of the set of mangrove-inhabiting species (*G. magnirostris*, *G. tenebrosa*, and *G. levigaster*). We also examine the biogeographic influence of island species and timing of speciation events tied to mangrove forests.

## Materials and Methods

### Taxon sampling and laboratory protocols

Our ingroup of 16 of the 19 *Gerygone* species comprised single samples per taxon and so was not designed to test species limits, which mostly are uncontroversial. We recognize that we are thus providing a framework with which later work can screen multiple samples for cryptic diversity and gain further evolutionary insight especially concerning some more geographically widespread (e.g., *G. fusca*, *G. sulphurea*) and naturally fragmented species (*G. levigaster*, *G. chloronota*). Unsampled taxa included the now extinct *G. insularis* of Lord Howe Island and extant populations of *G. dorsalis* and *G. albofrontata* from the Lesser Sundas and Chatham Islands, respectively. Outgroup taxa were chosen based on results of previous higher-level phylogenetic studies of passerines, and included diverse acanthizids: *Oreoscopus gutturalis* (Fernwren), *Smicrornis brevirostris* (Weebill), and *Acanthiza apicalis* (Inland Thornbill).

Genomic DNA was extracted from frozen or ethanol preserved tissue samples from vouchered specimens collected by us and researchers from other institutions (Table 1) via the standard Qiagen DNeasy™ tissue extraction protocols (Qiagen, Valencia, CA). We amplified and sequenced 13 distinct loci distributed across the avian nuclear and mitochondrial genomes using a published set of primers and protocols (Table 2). A detailed list of GenBank accession numbers for all loci and species is listed in Methods S1. All PCR amplifications were performed in 25 µl reactions using PureTaq™ RTG PCR beads (GE Healthcare Bio-Sciences Corp.). Amplified double-stranded PCR products were cleaned with ExoSAP-IT™ (GE Healthcare Bio-Sciences Corp.), and visualized on high-melt agarose gels stained with ethidium bromide. Purified PCR products were subsequently cycle-sequenced with ABI Prism BigDyeT™ v3.1 terminator chemistry using the same primers as for each PCR reaction. Cycle-sequenced products were further purified using Sephadex™ spin columns (GE Healthcare Bio-Sciences Corp.), and finally sequenced on an ABI 3130 automated sequencer. Sequences of both strands of each gene were examined and aligned in Sequencher 4.8 (GeneCodes Corp.). We did not attempt to reconcile the allelic phase of heterozygous base calls, but rather coded them as ambiguous according to the International Union of Pure and Applied Chemistry (IUPAC) standards. All sequences were deposited on GenBank under accession numbers JQ039483-JQ039727.

**Table 1:** Taxon sampling, voucher information, and locality information of *Gerygone* species included in the present study.

Taxon	Voucher	Locality
*Gerygone albogularis*	ANWC 26490	New Guinea, Central Province, Port Moresby
*Gerygone chloronota*	ANWC 39172	Australia, WA, Mitchell Falls
*Gerygone chrysogaster*	KUBI 7504	New Guinea, Western Province, Ekame Camp
*Gerygone cinerea*	KUBI 16404	New Guinea, Central Province, Mt. Simpson Bush Camp
*Gerygone flavolateralis*	AMNH DOT6559	Solomon Islands, Rennell Island, Tahamatangi
*Gerygone fusca*	ANWC 40265	Australia, NT, Kunoth Bore, NW of Alice Springs
*Gerygone igata*	MUNZ 12431	New Zealand, Palmerston North, Turitea Road
*Gerygone inornata*	WAM 23458	Indonesia, Sabu
*Gerygone levigaster*	ANWC 39335	Australia, QLD, SE of Gladstone
*Gerygone magnirostris*	ANWC 39961	Australia, QLD, N of Innisfail
*Gerygone modesta*	ANWC 40523	Australia, Norfolk Island Territory
*Gerygone mouki*	ANWC 39196	Australia, NSW, NNE of Kempsey
*Gerygone palpebrosa*	ANWC 39361	Australia, QLD, Miriam Vale
*Gerygone ruficollis*	ANWC 26963	New Guinea, Gulf Province, Mountain Camp
*Gerygone sulphurea*	AMNH DOT12621	Indonesia, Sulawesi, Bangai
*Gerygone tenebrosa*	ANWC 39184	Australia, WA, Point Torment
*Acanthiza apicalis*	ANWC 24367	Australia, QLD, S of Winton
*Smicrornis brevirostris*	ANWC 24332	Australia, NSW, NW of Cootamundra
*Oreoscopus gutturalis*	ANWC 39536	Australia, QLD, Longlands Gap, S of Atherton

Institutional abbreviations for voucher sources are as follows: American Museum of Natural History (AMNH), Australian National Wildlife Collection (ANWC), The University of Kansas Biodiversity Institute (KUBI), Massey University New Zealand (MUNZ), Western Australian Museum (WAM).

**Table 2:** Summary of the thirteen loci included in the present study.

Locus	Length(aligned bp)	Category, chromosome #^a^	Substitution model	A,C,G,T frequency	Variablesites (% total)	Informativesites (% total/% variable)	Source
MameAL-06	415	anonymous locus	TrN	0.267, 0.169, 0.270, 0.293	47 (11.32)	15 (3.61/31.91)	Lee and Edwards (2008) [55]
MameAL-16	387	anonymous locus	HKY+G	0.241, 0.230, 0.213, 0.314	66 (17.05)	24 (6.20/36.36)	Lee and Edwards (2008) [55]
MameAL-23	428	anonymous locus	TrN+I	0.324, 0.234, 0.177, 0.264	88 (20.56)	25 (5.84/28.40)	Lee and Edwards (2008) [55]
CDC132	597	intron, 2	TVM+G	0.264, 0.171, 0.216, 0.347	93 (15.57)	39 (6.53/41.93)	Backström et al. (2008) [56]
HMG2	494	intron, 4	TVM	0.314, 0.172, 0.203, 0.309	76 (15.38)	15 (3.03/19.73)	Backström et al. (2008) [56]
Fib5	621	intron, 4	HKY+G	0.299, 0.176, 0.201, 0.323	96 (15.46)	41 (6.60/42.70)	Marini and Hackett (2002) [57]
G3PDH	279	intron, 1	HKY	0.260, 0.337, 0.185, 0.216	37 (13.26)	9 (3.22/24.32)	Fjeldså et al. (2003) [58]
TGFb2	563	intron, 3	GTR+I	0.229, 0.243, 0.211, 0.315	105 (18.65)	33 (5.86/31.42)	Primmer et al. (2002) [59]
MUSK	560	intron, Z	HKY+I	0.298, 0.168, 0.194, 0.337	117 (20.89)	22 (3.92/18.80)	F.K. Barker (pers.comm.)
RAG1	1350	exon, 5	TrN+I+G	0.316, 0.219, 0.232, 0.232	108 (8.00)	41 (3.03/37.96)	Barker et al. (2002) [60]
RAG2	1038	exon, 5	HKY+I+G	0.289, 0.210, 0.238, 0.262	94 (9.05)	25 (2.40/26.04)	Barker et al. (2002) [60]
ND2	1041	mitochondrial	GTR+I+G	0.298, 0.389, 0.104, 0.206	359 (34.48)	255 (24.50/71.03)	Sorenson et al. (1999) [61]
ND3	351	mitochondrial	TrN+I+G	0.325, 0.361, 0.097, 0.215	133 (37.89)	86 (24.50/64.66)	Sorenson et al. (1999) [61]

a Locus information and chromosome number was inferred from the genome map of the chicken genome on GenBank.

### Data matrix construction and phylogenetic analyses

Complementary gene sequence contigs derived from all 13 loci for all taxa were aligned using ClustalX 2.0.7 [25], and scrutinized further by eye in Mesquite 2.74 [26]. Separate data matrices of 19 taxa (16 ingroup and 3 outgroup) were assembled for each of the 11 nuclear loci, while the two mitochondrial genes (ND2 and ND3) were combined in a single dataset. Subsequent analyses examined individual loci and a partitioned dataset through model-based phylogenetic algorithms under both Maximum Likelihood (ML) and Bayesian analysis (BA) approaches. ModelTest 3.7 [27] was used to determine the most appropriate model of sequence evolution via the Akaike Information Criterion (AIC).

ML heuristic tree searches were conducted using the program GARLI 2.0 [28], under a single data partition and the GTR+I+G model of sequence evolution as well as partitioned by locus with the respective models of evolution and parameter values estimated from the data. Two separate runs were performed and nodal support was assessed via 1000 non-parametric bootstrap replicates. BA was carried out within the Markov Chain Monte Carlo (MCMC) tree search algorithm framework as implemented in the program MrBayes 3.1.2 [29]. The concatenated data set was partitioned by each locus, and by codon position for the mitochondrial genes. We ran two independent runs of 10^7^ generations, using the previously inferred model of sequence evolution specified for each locus. Search parameters included unlinking of all partition-specific rates and models of evolution, adjustment of chain heating conditions (temp = 0.1–0.05) for improved chain swap acceptance rates, and sampling every 100 generations. Evaluation of stationarity and chain convergence was conducted by plotting posterior probabilities from the two runs in the program Tracer [30]. The resulting pool of topologies sampled from the first 30% of generations of each of the two independent runs was discarded as an initial ‘burn-in’, and the resulting pool of trees from both runs were finally summarized to produce a single 50% majority-rule consensus tree, rooted with the Fernwren *Oreoscopus gutturalis*. Lastly, we proceeded to evaluate the monophyly of the 3 mangrove-restricted gerygones by enforcing their monophyly as a constraint on ML GARLI searches. Site likelihood outputs from the best constrained trees were used in subsequent test against our ML tree via the Approximately Unbiased (AU) test, as implemented in the program CONSEL [31].

Additionally, a species tree was estimated from the joint distribution of individual gene trees via the program BEST 1.6 [32], [33]. The dataset was again partitioned by locus, each with an appropriately specified model of evolution. We assigned default settings for the parameter values of the Bayesian search, as recommended by the authors: flat priors, inverse gamma distribution with values of α = 3 and β = 0.003 for priors of population size, and a uniform distribution with bounds of 0.5 and 1.5 for priors of the mutation rates. Two runs with four separate chains (one heated and three cold) were run simultaneously for 10^8^ generations, sampling every 1000 generations. A consensus topology from the two separate runs was obtained after discarding an initial burn-in of 50% of the sampled topologies. Additionally, we also used the species tree reconstruction options in the program *BEAST 1.6 [34], [35] using the same set of model parameterizations and number of generations as for the BEST run.

### Phylogenetic affinities and timing of divergence of *G. cinerea*

Initial examination of the data revealed that sequences of the Grey Gerygone, *G. cinerea*, from the highlands of New Guinea were substantially distinct from other *Gerygone* species. This prompted us to consider further testing of the phylogenetic placement of *G. cinerea* within the Meliphagoidea in which *Gerygone* itself is embedded. Gardner et al. 's (2010) study of Meliphagoidea shared three markers with our dataset. Accordingly, we assembled a separate data matrix from published and newly derived sequences for nuclear exons of RAG1 and RAG2 and the mtDNA gene ND2 to examine relationships of *G. cinerea* within the Acanthizidae specifically and Meliphagoidea more generally (Methods S1).

We performed a Bayesian analysis using the program MrBayes 3.1.2 as described above, partitioning our data by gene and by codon for the two nuclear and the mitochondrial genes, respectively. This larger dataset was also used to estimate relative timing events of cladogenesis using the program BEAST 1.6 [34] by producing an ultrametric tree with 95% confidence intervals for node heights. Given the lack of reliable fossil calibration points for acanthizids, we placed a broad normal distribution (2.0×10^−8^±3.5×10^−9^ substitutions/site/year) on the ND2 mutation rate prior, while the RAG genes were parameterized with a broader lognormal prior. This range encompasses previously published passerine mitochondrial rates of evolution based on calibrations using a combination of fossil and biogeographic dates [36]–[38]. A topological constraint in the form of the Bayesian consensus tree was placed onto the MCMC run, such that rates were allowed to vary only along this given scenario. A relaxed clock model [39] with uncorrelated rates drawn from a lognormal distribution was selected, and two MCMC runs of 10^7^ generations with parameters logged every 100 generations were performed. The first 40% of generations of each run were discarded as burn-in after inspection of likelihood scores and parameters for stationarity. The final ultrametric tree was generated from the combined tree files of the two MCMC runs.

## Results

### Phylogenetic analyses of gene trees and species tree reconstruction

Alignment of sequence data derived from all thirteen loci was straightforward, resulting in a total of 8124 base pairs (bp). Overall sequence length ranged from 279 bp to 1350 bp for nuclear loci, whereas the two mitochondrial genes were 1041 bp and 351 bp in length (Table 2). Among the nuclear loci, MameAL-23, MUSK, and TGFb2 were the most variable; however, MameAL-16, CDC132 and Fib5 had the highest percentage of informative sites (Table 2). The two mtDNA protein-coding genes ND2 and ND3 had no insertions, deletions, or anomalous stop-codons. Base composition was typical of avian mtDNA (Table 2), consistent with true mitochondrial origin as opposed to nuclear pseudogenes [40]. Information content in the two mitochondrial loci was significantly higher than in the nuclear loci: out of the total number of variable sites, ND2 and ND3 had over 70% and 64% parsimony informative sites, respectively (Table 2).

**Figure 1:**
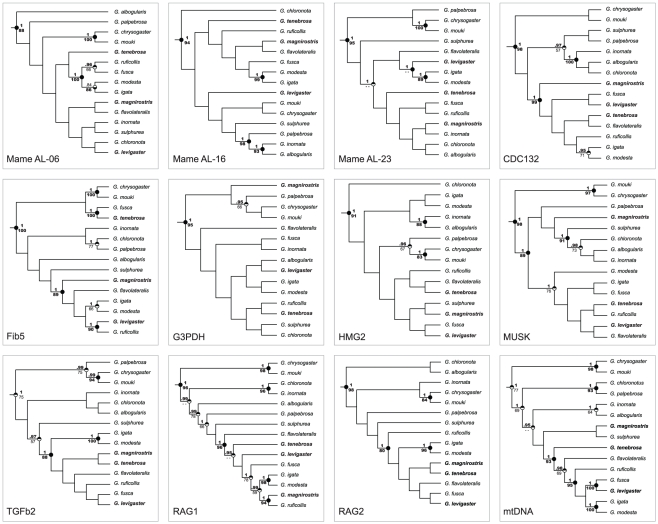
Phylogenetic estimates of gene trees obtained via Bayesian and Maximum Likelihood analysis of individual loci. Locus acronyms follow Table 2 and references therein. Nodal support is indicated by circles, where the upper half corresponds to Bayesian posterior probabilities (BPP) and the lower half depicts ML bootstrap values (MLBV). BPP support values greater than 95% are given in bold above branches, and indicated by dark upper half-circles. MLBV greater than 80 are in bold below branches, and indicated by dark lower half-circles. Support values below these thresholds are in regular font and depicted with an open circle half. Values below 50% BPP and 50 MLBV are denoted by double dashes or not at all where both algorithms failed to recover that value at a node. The mitochondrial protein coding genes ND2 and ND3 have been combined in a single partition, indicated as “mtDNA”. Mangrove specialists are highlighted in bold.

**Figure 2:**
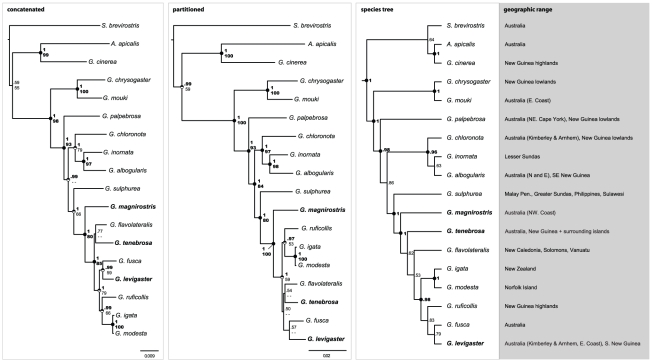
Phylogenetic analyses of the combined 13-locus dataset. All topologies are rooted with the Fernwren *Oreoscopus gutturalis* (not shown for brevity). Support values in form of Bayesian posterior probabilities (BPP) and Maximum Likelihood bootstrap (MLBV) are given above and below each node, respectively, with dark circles and bold font emphasizing strong support (>95% BPP and >80 MLBV). Regular font and open circle halves depict support values below these thresholds. A double dash depicts support values below 50% BPP and 50 MLBV. The concatenated phylogenetic hypothesis in the left panel is based on analyses of the entire dataset under a single, concatenated partition. The center panel represents the topology derived from an analysis of the entire dataset partitioned by locus and codon position for the two mitochondrial protein coding genes. The topology in the right panel illustrates the species tree obtained under the BEST algorithm. Mangrove specialists are highlighted in bold. Geographic range is given alongside taxa of the species tree.

Resolution of individual gene trees varied at diverse nodes throughout their topologies, most loci showing consistent patterns of sister species relationships (Figure 1). G3PDH was the least informative locus and also the shortest sequence, but all other nuclear loci showed at least four strongly supported nodes (Figure 1). The combined mitochondrial dataset (ND2 and ND3) featured the best-resolved topology, and all but three nodes received high support. Analysis of the concatenated dataset under a single partition and partitioned by gene and codon region for the two mtDNA protein-coding genes recovered similar topologies and statistical support to our species tree estimate (Figure 2, see below). Nodal support was strong throughout the concatenated and partitioned datasets: only some terminal nodes received relatively low statistical support (Figure 2). Compared to the species tree estimate, the concatenated and partitioned datasets differed in placement of *G. tenebrosa* relative to *G. flavolateralis*, a relationship that has seen generally weak support. Further differences are also evident along subsequent nodes, although the three different data analysis schemes agreed on the majority of the relationships except for the most recent speciation events.

*G. cinerea* was consistently recovered by all loci as not closely related to other ingroup species, rendering *Gerygone* paraphyletic (Figure 1, 2). Analysis of our 13-locus dataset placed this species among the three outgroup members, and specifically with the species we used of *Acanthiza*, *A. apicalis*.

**Figure 3:**
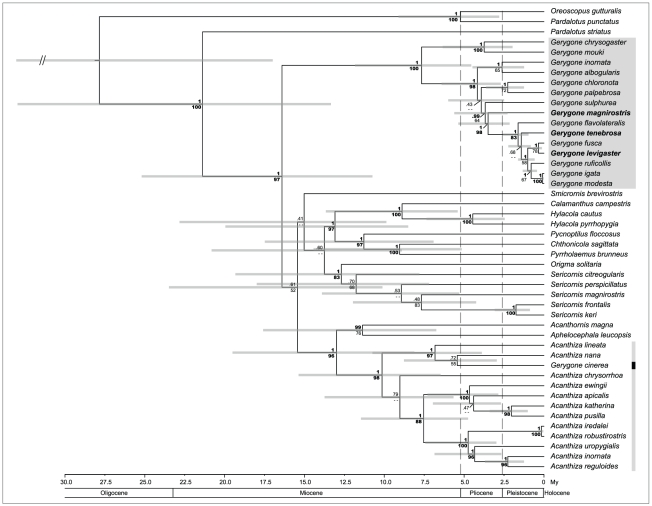
Phylogenetic hypothesis of relationships within the broader family Acanthizidae. Results are based on a three gene dataset (RAG1, RAG2, ND2) with extended taxon sampling derived from the study of Gardner et al. (2010). Nodal support in form of Bayesian posterior probabilities (BPP, above) and Maximum Likelihood bootstrap values (MLBV, below) are given at each node. Bold values are attributed to BPP >95% and MLBV >80, while regular font is used for values below this threshold. A double dash indicates support values below 50% BPP and 50 MLBV. Also illustrated are 95% confidence intervals around node heights as derived from the ultrametric tree generated in the program BEAST and calibrated using a normally distributed prior on ND2 mutation rates. The lower scale represents time in million years before present, and vertical dashed lines correspond to the onset of the Pliocene and Pleistocene epochs. Mangrove specialists are highlighted in bold. Placement of *Gerygone cinera* within *Acanthiza* is emphasized by a black bar.

We pursued the phylogenetic placement of *G. cinerea* within acanthizids generally by using the three gene dataset assembled with broad taxon sampling of the Meliphagoidea (see Methods S1). The dataset comprised 3429 bp from RAG1 (1350 bp), RAG2 (1038 bp) and ND2 (1041 bp) (Methods S1). Results clearly reinforced our previous inferences based on the 13-locus dataset that *G. cinerea* clustered not with *Gerygone* but with *Acanthiza*, the second largest genus of acanthizid warblers. Placement of *G. cinerea* within *Acanthiza* received strong nodal support (Figure 3): within *Acanthiza*, *G. cinerea* is most closely related to *A. lineata* and *A. nana* of Australia and *A. murina*, which until now was thought to be the only species of *Acanthiza* in New Guinea (see Nicholls *et al.* 2000) [41].

All gene trees indicated clearly that the three mangrove-inhabiting species *G. magnirostris*, *G. tenebrosa*, and *G. levigaster*, do not form a monophyletic group. Strong support was evident in all gene trees for two sister species relationships, one between *G. chrysogaster* and *G. mouki*, and the other between *G. igata* and *G. modesta*. The mtDNA dataset further indicated strong support for sister species relationships between *G. chloronota* and *G. palpebrosa* (also supported by Fib5), between *G. inornata* and *G. albogularis* (also supported by MUSK, HMG2, AL16), and between *G. fusca* and *G. levigaster* (also supported by RAG2, TGFb2, HMG2, CDC132).

The species tree inferred from all 13 loci mirrored closely the consensus among the underlying gene trees and the analysis of the concatenated and partitioned dataset. Topologies obtained throught the BEST and *BEAST algorithms were congruent. Again, *Gerygone* was not monophyletic and the sister species relationships of *G. chrysogaster/G. mouki*, and *G. igata/G. modesta* were strongly supported (Figure 2). Similarly, the three mangrove specialists were not a monophyletic group, and their constrained monophyly constitutes a significantly worse likelihood under the AU test. The majority of nodes in the species tree received strong support; however, several low-to-moderately supported nodes prevailed, especially in the recently evolved clades sister to *G. magnirostris*.

### Timing of speciation events

The same extended dataset was used to infer a sequence of splitting events under a relaxed-clock model coupled with an enforced topological constraint from the Bayesian consensus tree. The resulting ultrametric tree illustrates important variation in the 95% confidence intervals for node heights (Figure 3). As such, we can clearly distinguish differences in evolutionary rates between the two most speciose acanthizid genera, *Gerygone* and *Acanthiza*, the former clearly having radiated later around the onset of the Pliocene, and with increased speciation rate, whereas the clade containing *Acanthiza*, *Sericornis*, and other Australo-Papuan acanthizids is relatively older, stemming well into the Miocene and has had slower rates of diversification. Based on uncorrected sequence divergences of the two mitochondrial genes, the genetically most distinct gerygones (excluding *G. cinerea*) were *G. palpebrosa* and *G. mouki* at 13.5%. Highest divergence values within the clade containing the three mangrove specialist species (Figure 3) were at 8.1% between *G. magnirostris* and *G. igata*. The three mangrove endemics differed by 7.7% (*G. magnirostris* vs. *G. tenebrosa*), 7.3% (*G. magnirostris* vs. *G. levigaster*), and 4.0% (*G. levigaster* vs. *G. tenebrosa*).

## Discussion

### Multilocus phylogenetic analysis and taxonomy of *Gerygone*

Our study represents the first comprehensive phylogenetic analysis of the acanthizid warbler genus *Gerygone* and we have used a broadly sampled, multilocus dataset. While multilocus phylogenetic analyses have been successfully employed throughout a diverse array of avian groups [13], [42]–[46], the present study explored the utility of a moderate number of unlinked loci spread across the avian nuclear and mitochondrial genomes to better understand the implications of individual gene histories and their influence on species tree estimation. [17], [20], [21], [47]. Moreover, we focused on a group having diverse evolutionary and ecological histories. Overall, several common phylogenetic patterns emerged from the individual gene trees but their differences also highlight complexity in the group's evolutionary history. The Bayesian estimate of species tree relationships and the analyses of the concatenated and partitioned dataset resulted in very similar topologies. Below, we highlight details of some of these commonalities and differences among analytical methods.

The most novel relationship that we recovered is the exclusion from *Gerygone* of *G. cinerea*, which clearly belongs in *Acanthiza* (Figure 2 and 3). Based on plumage and biogeography, Ford (1986) suggested that *G. cinerea* was closely related to *G. chloronota*. We conclude that *G. cinerea* should be assigned to *Acanthiza* Vigors and Horsfield, 1827, and so be known as *A. cinerea* (Salvadori, 1876).

Ford's [1] taxonomic study of *Gerygone* based on numerical analysis of morphological characters noted inherent difficulties in reconstructing relationships based solely upon morphology. It nevertheless derived important hypotheses regarding sister species relationships of gerygones, some of which were corroborated here by multilocus data. For example, two relationships suggested by Ford [1], that of *G. inornata* of the Lesser Sundas being closely related to Australo-Papuan *G. albogularis*, and Australian *G. fusca* being closely related to the mangrove forest endemic *G. levigaster*, were affirmed here in the species tree, three of the gene trees, and the mtDNA tree (Figure 1 and 2). Further, the hypothesis that eastern Australian endemic *G. mouki* is a basal member of the gerygones [1], [48], was supported almost unequivocally in our different data analyses (Figures 1, 2, and 3).

Other novel relationships within *Gerygone* include the eastern Australian endemic *G. mouki* as sister to *G. chrysogaster* from the lowlands of New Guinea, and the grouping of *Gerygone chloronota* with *G. inornata* and *G. albogularis* (Figure 2). Another unequivocally supported sister species relationship was between the endemics of New Zealand and Norfolk Island, *G. igata* and *G. modesta*, respectively. Ford [1] had alternatively concluded that *G. modesta* and *G. igata* are not sister taxa and that the former is possibly more closely related to mangrove-restricted *G. levigaster*. Nonetheless, our analyses and earlier ones [1], [48] affirm that *G. levigaster* is close to *G. fusca*, which is widespread on the Australian continent.

Several *Gerygone* species were characterized by weakly-supported phylogenetic placements in the species tree analysis. Low nodal support was present at more recent radiations in clades sister to *G. magnirostris*. As such, phylogenetic uncertainties remain about the position of the New Guinean montane endemic *G. ruficollis*. The species tree places it with low support as sister to the *G. fusca*/*G. levigaster* pair (Figure 2), but the concatenated and partitioned dataset analysis instead supported it as sister to *G. igata/G. modesta* (Figure 2). Interestingly, our mtDNA dataset includes *G. ruficollis* as sister to a clade containing both of these other sister species pairs. Also weakly resolved was the phylogenetic placement of the Pacific Island *G. flavolateralis* as sister to the Australian mangrove endemic *G. tenebrosa* in the concatenated and combined analyses; in contrast, the species tree reconstruction did not recover a direct sister species relationship.

### Biogeographic patterns and the evolution of mangrove-restricted gerygones

Complex evolutionary and biogeographic scenarios in the history of *Gerygone* are clearly apparent from our results. They identified *G. chrysogaster* and *G. mouki* as a sister clade to the rest of *Gerygone*, consistent with an Australo-Papuan center of diversity for the group. The geographic distributions of these two taxa correspond to Australo-Papuan tropical lowland (Irian) and subtropical-montane rainforest (Tumbunan) avifaunas [3], [5], [49].

The clade formed by *G. chloronota* as sister to *G. inornata* and *G. albogularis* includes species from northwest Australia and New Guinea, the Lesser Sundas, northeast Australia and southeast New Guinea, respectively. The sister relationship between insular *G. inornata* and continental *G. albogularis* likely reflects either vicariance, probably by rising sea-level across Torres Strait and the Arafura Platform, or dispersal across the same region in the history of speciation within this clade between Australian and New Guinean landmasses [5], [50]. The only *Gerygone* species that extends beyond Wallace's Line, *G. sulphurea*, has radiated into the Malay Peninsula, Greater Sundas, and the Philippines, where it occupies forests as well as coastal mangroves. The lone position of this geographically wide-ranging species in the phylogeny on a long branch amidst different subclades of gerygones is notable (Figure 2 and 3). Given that the Acanthizidae generally are sedentary, we suggest that an ecological study of this species and another wide-ranging species such as Australian *G. fusca* in conjunction with refined knowledge of their phylogenetic position based on more extensive population sampling of each would be rewarding.

The remaining species of *Gerygone* are from continental Australia, New Guinea, and islands of the Pacific Ocean (Figure 2). Prominent in this group are the three mangrove-inhabiting species *G. magnirostris*, *G. tenebrosa*, and *G. levigaster*. Despite some residual phylogenetic uncertainty, particularly concerning the north-west Australian endemic *G. tenebrosa*, our data do show that these three species do not represent a single radiation in mangrove ecosystems. Rather, they appear to represent two if not three independent events of adaptive colonization of mangroves, whether derived from continental or island sister species. Further, our analysis indicates that for these three species mangroves were first colonized by the lineage that evolved into *G. magnirostris*, then by that which evolved into *G. tenebrosa* and, finally, that for *G. levigaster* (Figure 2 and 3). This provides a historical framework within which to pursue the evolution of their different habitat preferences and bill morphologies, and the extent and patterns of their geographical range overlaps, especially in north-western Australia. These patterns and overlaps have been detailed extensively for that region [2], [51], [52]. For example, *G. magnirostris* and *G. levigaster* overlap more extensively than do *G. levigaster* and *G. tenebrosa*, whereas *G. magnirostris* and *G. tenebrosa* barely overlap. *G. levigaster*, which is more closely related to a species widespread in inland Australia (*G. fusca*) than to *G. magnirostris* and *G. tenebrosa*, inhabits mangroves almost exclusively dominated by *Avicennia* and *Ceriops* species and *Melaleuca* thickets. *G. tenebrosa* inhabits mangrove forests, woodlands and thickets of *Avicennia*, *Bruguiera*, *Camptostemo*n and *Ceriops*, and *G. magnirostris* prefers taller *Rhizophora* and *Bruguiera* stilt-rooted mangroves. *G. magnirostris* also inhabits swamplands and riparian forests adjacent to its main, mangrove-preferred habitat [2], [51], [52].

Sequence divergences and timing estimates based on ND2 mutation rates suggest a more recent evolution of *Gerygone* with respect to other members of the acanthizid clade (Figure 3). Some degree of past or present hybridization between taxa such as *G. magnirostris* and *G. tenebrosa* [53], [54], which can complicate species tree inferences, may also be involved. Concerning the temporal framework of speciation in *Gerygone*, it is clear that it was relatively quick, and originated in the late Miocene, with most cladogenetic events within *Gerygone* occurring in the Pliocene and Pleistocene (Figure 3). This is supported by the lack of consensus in phylogenetic resolution of most relevant taxa (Figure 2 and 3). Thus, all three methods we have used had difficulties recovering a well supported evolutionary pattern for the most recent clades. Variable placements of the Pacific Islands endemic *G. flavolateralis* and the New Guinean montane endemic *G. ruficollis* all illustrate this. Multilocus phylogenetic analysis has seen a surge of attention in recent years, although difficulties associated with obtaining well-supported phylogenetic topologies from such a large and diverse array of loci can lead to a sense of low return given the considerable effort required for generating such datasets. Differences in topologies and support can derive from difficulties in proper parameterization of models applied to such large datasets, further complicated by rapid rates of speciation over broad geographic scales and ecological niches. We are, however, certain that such repeated efforts in generating well-sampled datasets for non-model organisms will lead to an increased understanding of their complex evolutionary histories. We should be prepared to recognize that sometimes different facets of one biological question may be answered by different elements of a data set as mitochondrial and nuclear DNA data have done here. Conversely, understanding when to build or not build more complex datasets should always remain an important element that guides how one answers a particular question.

Thus, gerygones colonized mangroves on several occasions and those that occur in mangroves are not each other's closest relatives within the genus *Gerygone*. This lends further support for case-by-case exploration of the rich Australo-Papuan mangrove avifauna. Phylogeographic analysis of diversity within and among the three gerygones adapted to mangroves and their closest relatives especially *G. fusca*, will bring additional insights to levels of intraspecific genetic diversity, influence of geographic barriers, and the history of any hybridization events. Contrasting these molecular findings with data based on morphology, plumage, song and ecological niche will broaden our understanding of historical biogeography within this group. In particular, it should clarify the importance of the mangroves of Australia and New Guinea in the evolution of the region's avifauna and its ecological diversity.

## Supporting Information

Methods S1 **Extended taxon sampling included in the analysis of** ***G. cinerea*** **within the Acanthizidae.** All samples are listed in Gardner et al. (2010) and include GenBank accession numbers from multiple sources used in building a multilocus dataset for testing relationships within the Meliphagoidea.
(DOC)
