# Correction: Evolution in Australasian Mangrove Forests: Multilocus Phylogenetic Analysis of the *Gerygone* Warblers (Aves: Acanthizidae)

**DOI:** 10.1371/annotation/2e9dfd8d-413c-47bd-84c2-d7df5db7859c

**Published:** 2012-06-07

**Authors:** Árpád S. Nyári, Leo Joseph

There was an error in Figure 3. The correct Figure 3 can be viewed here: 

**Figure:**
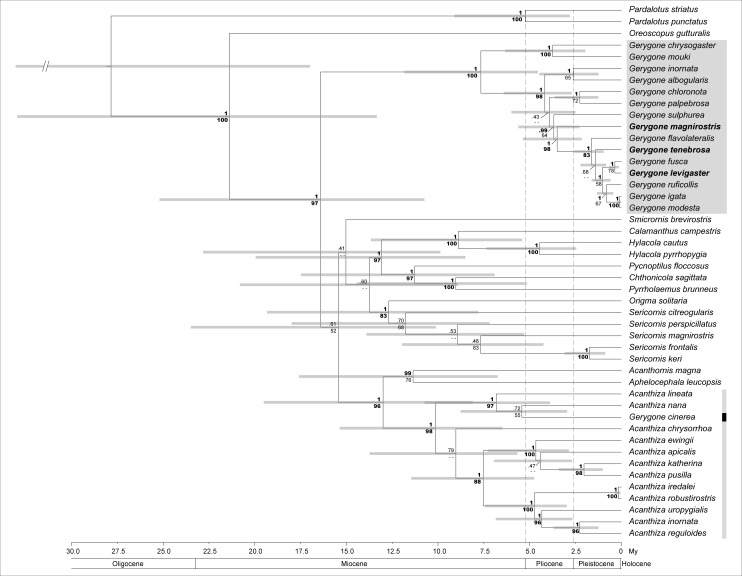


[^] 

