# Supplementary material for: Evolution in Australasian Mangrove Forests: Multilocus Phylogenetic Analysis of the Gerygone Warblers (Aves: Acanthizidae)
Source: PLoS One. 2012 Feb 14;7(2):e31840. doi: 10.1371/journal.pone.0031840 (PMC3280719; doi:10.1371/journal.pone.0031840)
Supplement: Methods S1 — Extended taxon sampling included in the analysis of G. cinerea within the Acanthizidae. All samples are listed in Gardner et al. (2010) and include GenBank accession numbers from multiple sources used in building a multilocus dataset for testing relationships within the Meliphagoidea. (DOC) [file pone.0031840.s001.doc]

| Taxon | RAG1 | RAG2 | ND2 |
| --- | --- | --- | --- |
| *Pardalotus punctatus* | GU825809 | GU825843 | AY488321 |
| *Pardalotus striatus* | GU825810 | GU825844 | AY488322 |
| *Calamanthus campestris* | GU825820 | GU825854 | GU825887 |
| *Hylacola cautus* |  | GU825856 | GU825889 |
| *Hylacola pyrrhopygia* | GU825855 | GU825821 | GU825888 |
| *Pycnoptylus floccosus* | GU825812 | GU825846 | GU825879 |
| *Chtonicola sagittata* | GU825819 | GU825853 | GU825886 |
| *Pyrrholaemus brunneus* | GU825818 | GU825852 | GU825885 |
| *Origma solitaria* | GU825813 | GU825847 |  |
| *Sericornis citreogularis* | GU825815 | GU825849 | GU825881 |
| *Sericornis perspicillatus* |  |  | AY488324 |
| *Sericornis magnirostris* |  |  | GU825883 |
| *Sericornis frontalis* | GU825816 | GU825850 | AY488323 |
| *Sericornis kerri* |  |  | GU825882 |
| *Acanthornis magna* | GU825817 | GU825851 | GU825884 |
| *Aphelocephala leucopsis* | GU825832 | GU825869 | GU825903 |
| *Acanthiza lineata* | GU825828 | GU825864 | GU825898 |
| *Acanthiza nana* | GU825827 | GU825863 | GU825897 |
| *Acanthiza chrysorrhoa* | GU825825 | GU825861 | AY488317 |
| *Acanthiza ewingii* |  |  | GU825892 |
| *Acanthiza katherina* |  |  | GU825890 |
| *Acanthiza pusilla* | GU825822 | GU825857 | GU825891 |
| *Acanthiza irendalei* |  |  | GU825895 |
| *Acanthiza robustirostris* |  |  | GU825899 |
| *Acanthiza uropygialis* | GU825826 | GU825862 | GU825896 |
| *Acanthiza inornata* | GU825824 | GU825860 | GU825894 |
| *Acanthiza reguloides* | GU825823 | GU825859 | GU825893 |
